# Galvanic Obstetric Forceps

**Published:** 1840-01

**Authors:** 

**Affiliations:** Bonn.


*Galvanic Obstetric Forceps.* By Prof. Kilian, of Bonn.

Desirous of ascertaining how forceps, the blades of which were made of different metals, would affect the uterus in tardy labors, Professor Kilian caused such a pair to be manufactured. An opportunity soon occurred for testing its efficacy. Labor was proceeding slowly from deficient action of the uterus, and for two hours and a half the head had remained in the same spot. The forceps were applied, and, as soon as the two blades were joined, the uterus was felt to contract powerfully, but not in such a manner as to assist in the expulsion of the child.

[Was not this mere accident?]—*Medicinische Zeitung.* No. xii. 1839.

